# Author Index

**DOI:** 10.1038/sj.bjc.6600443

**Published:** 2002-06-17

## Abstract

*British Journal of Cancer* (2002) **86**, 1973–1980. DOI: 10.1038/sj.bjc.6600443 www.bjcancer.com

© 2002 Cancer Research UK

